# Supplementary figures and images for: Effect of vascular endothelial growth factor 165 on dopamine level in the retinas of guinea pigs with form-deprivation myopia
Source: PeerJ. 2023 Oct 13;11:e16255. doi: 10.7717/peerj.16255 (PMC10578302; doi:10.7717/peerj.16255)

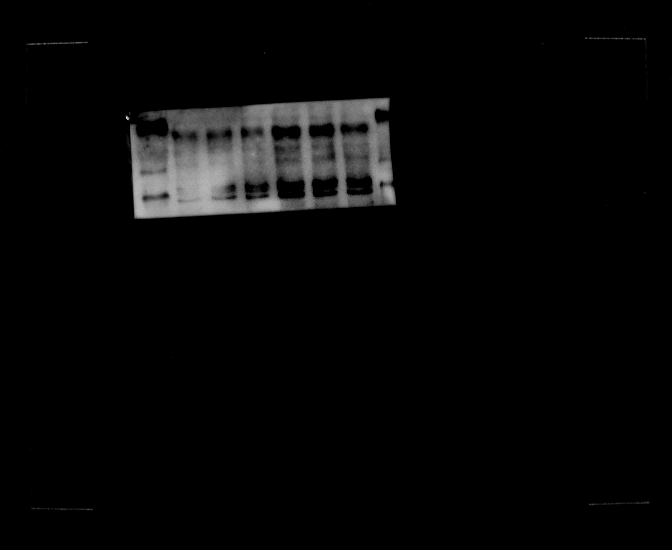

Supplement: Supplemental Information 2 [file peerj-11-16255-s002.zip › WB/CD31/CD1/c1.tif]

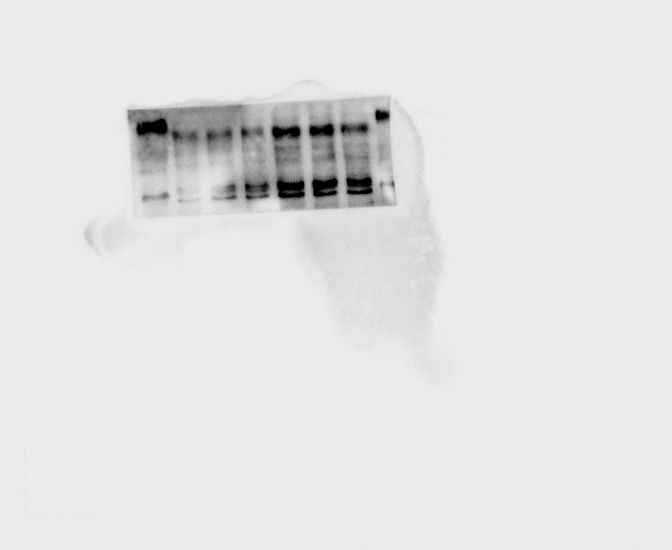

Supplement: Supplemental Information 2 [file peerj-11-16255-s002.zip › WB/CD31/CD1/c2.tif]

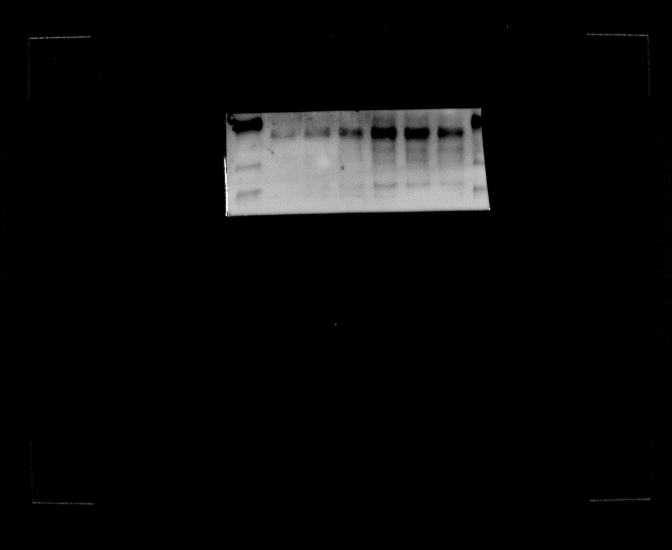

Supplement: Supplemental Information 2 [file peerj-11-16255-s002.zip › WB/CD31/CD2/c1.tif]

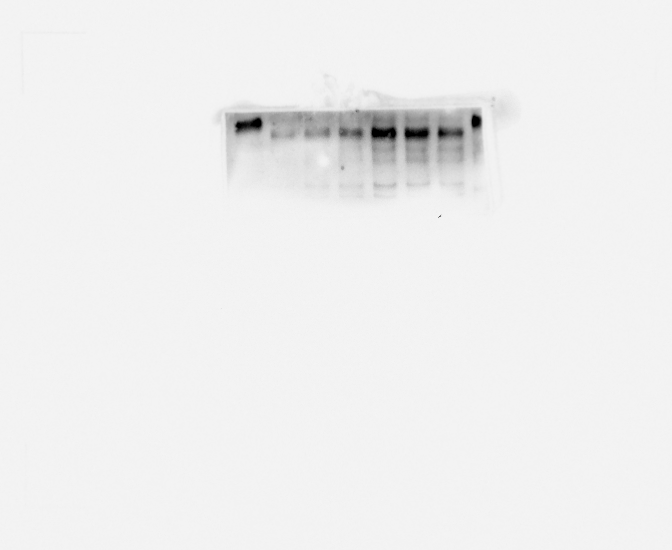

Supplement: Supplemental Information 2 [file peerj-11-16255-s002.zip › WB/CD31/CD2/c2.tif]

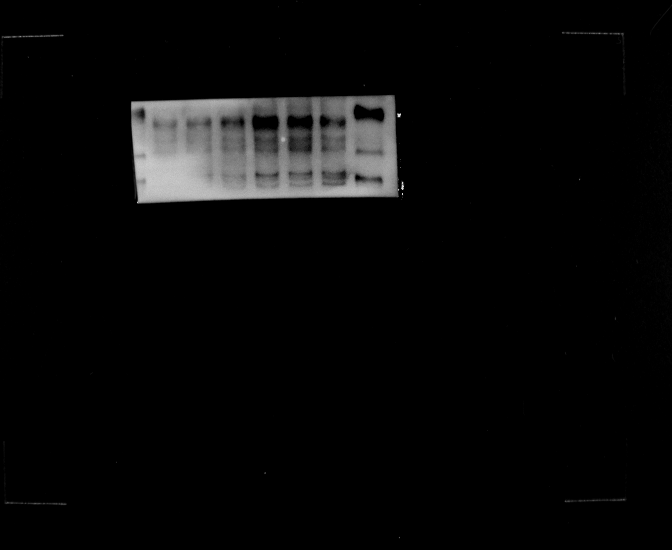

Supplement: Supplemental Information 2 [file peerj-11-16255-s002.zip › WB/CD31/CD3/c1.tif]

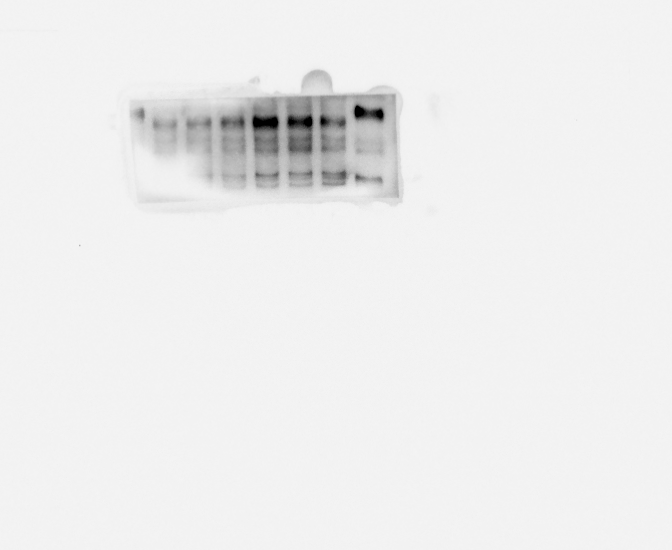

Supplement: Supplemental Information 2 [file peerj-11-16255-s002.zip › WB/CD31/CD3/c2.tif]

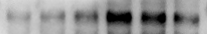

Supplement: Supplemental Information 2 [file peerj-11-16255-s002.zip › WB/CD31/CD3/c3.tif]

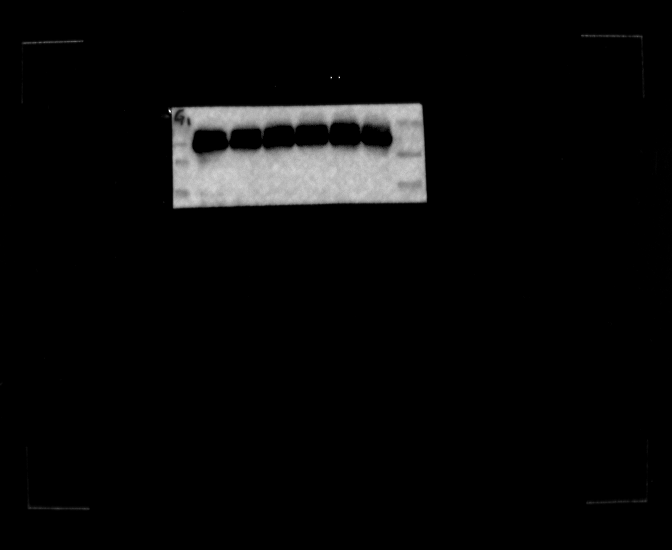

Supplement: Supplemental Information 2 [file peerj-11-16255-s002.zip › WB/CD31/GAPDH1/G1.tif]

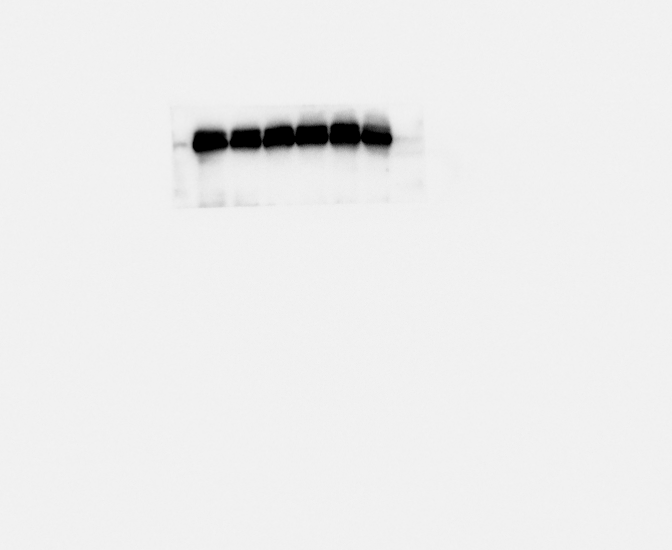

Supplement: Supplemental Information 2 [file peerj-11-16255-s002.zip › WB/CD31/GAPDH1/G2.tif]

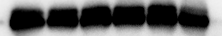

Supplement: Supplemental Information 2 [file peerj-11-16255-s002.zip › WB/CD31/GAPDH1/G3.tif]

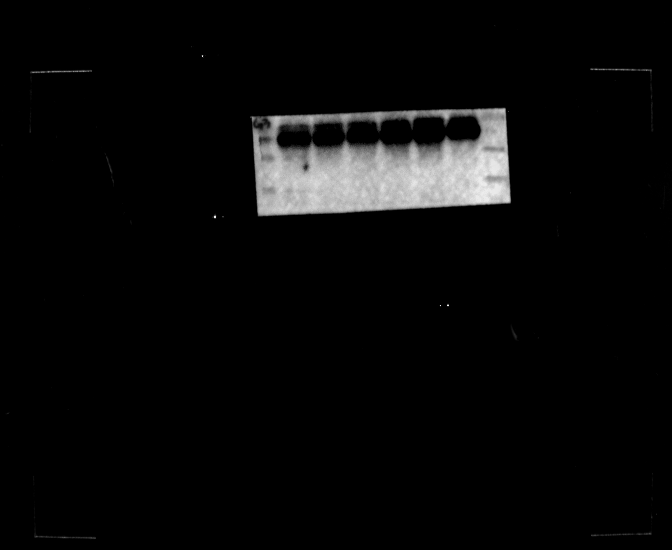

Supplement: Supplemental Information 2 [file peerj-11-16255-s002.zip › WB/CD31/GAPDH2/G1.tif]

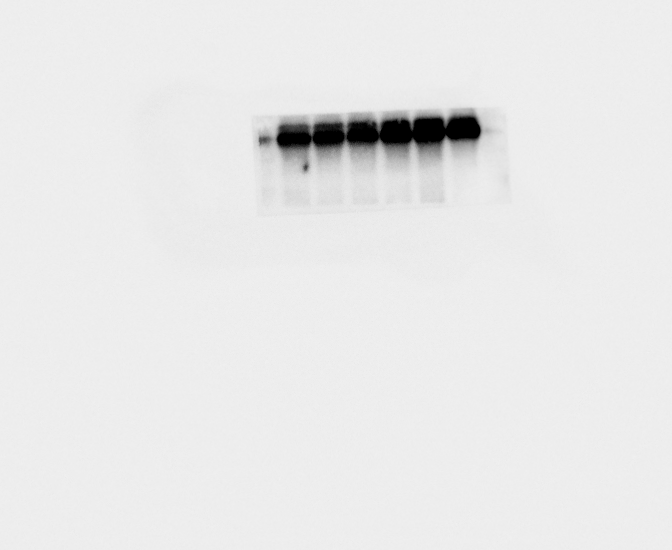

Supplement: Supplemental Information 2 [file peerj-11-16255-s002.zip › WB/CD31/GAPDH2/G2.tif]

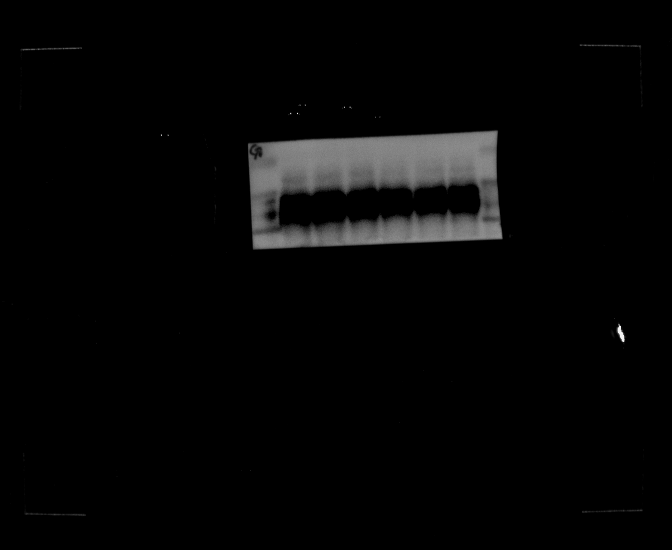

Supplement: Supplemental Information 2 [file peerj-11-16255-s002.zip › WB/CD31/GAPDH3/G1.tif]

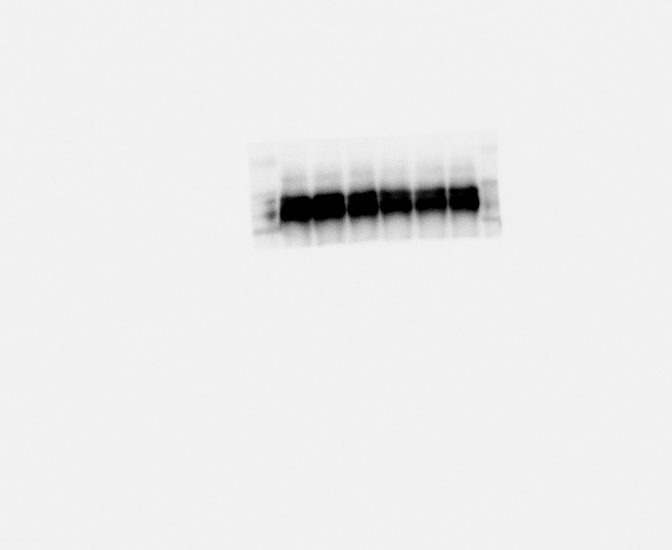

Supplement: Supplemental Information 2 [file peerj-11-16255-s002.zip › WB/CD31/GAPDH3/G2.tif]

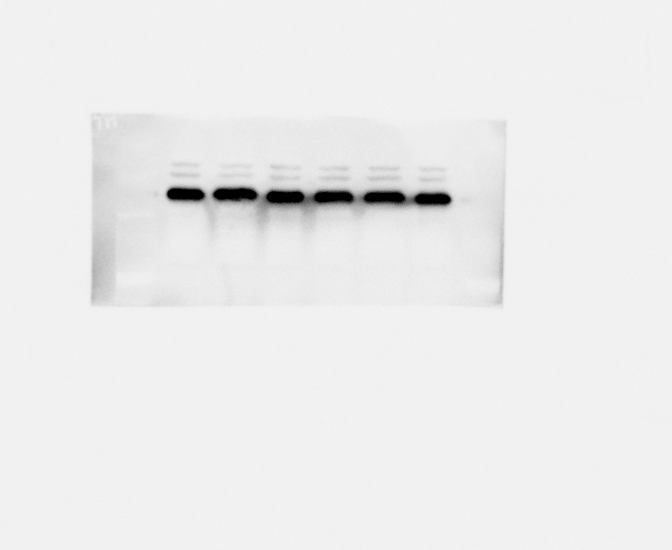

Supplement: Supplemental Information 2 [file peerj-11-16255-s002.zip › WB/TH/GAPDH1/G1.tif]

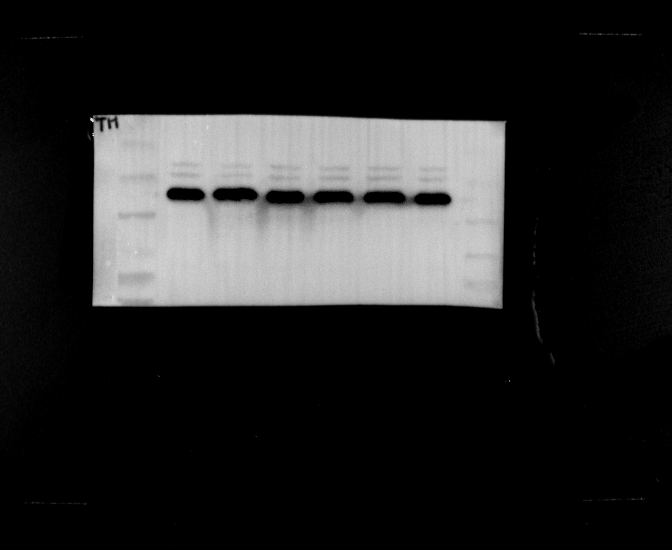

Supplement: Supplemental Information 2 [file peerj-11-16255-s002.zip › WB/TH/GAPDH1/G2.tif]

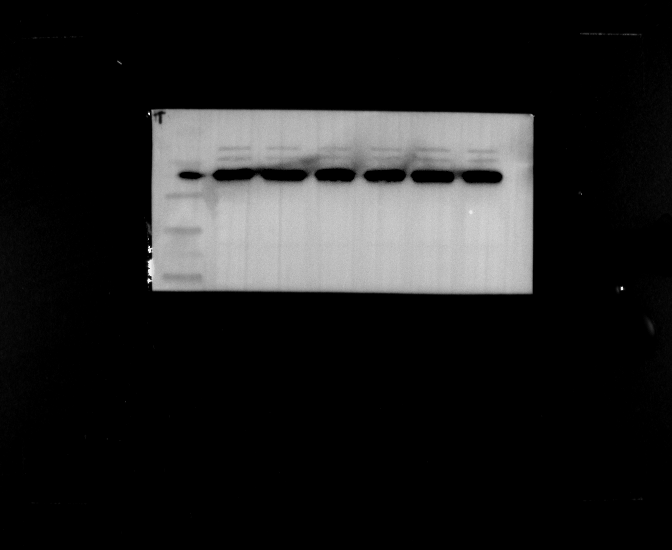

Supplement: Supplemental Information 2 [file peerj-11-16255-s002.zip › WB/TH/GAPDH2/G1.tif]

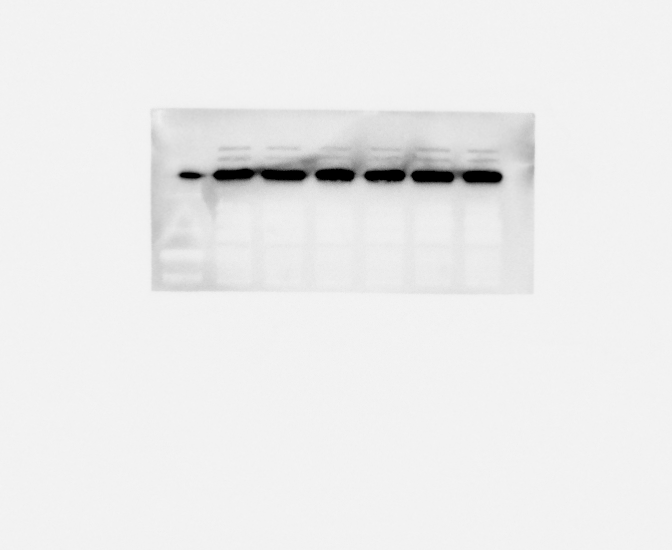

Supplement: Supplemental Information 2 [file peerj-11-16255-s002.zip › WB/TH/GAPDH2/G2.tif]

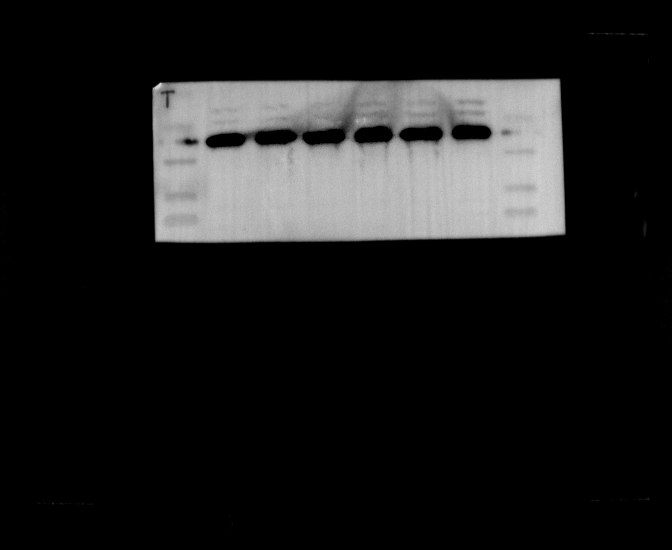

Supplement: Supplemental Information 2 [file peerj-11-16255-s002.zip › WB/TH/GAPDH3/G1.tif]

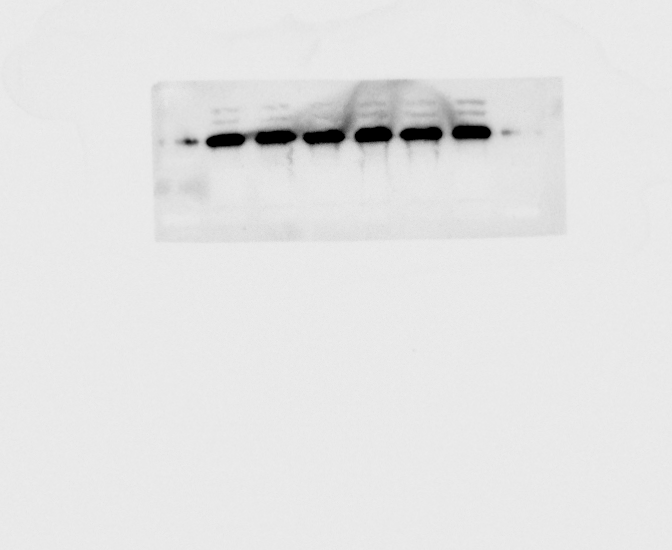

Supplement: Supplemental Information 2 [file peerj-11-16255-s002.zip › WB/TH/GAPDH3/G2.tif]

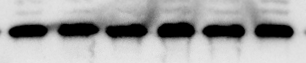

Supplement: Supplemental Information 2 [file peerj-11-16255-s002.zip › WB/TH/GAPDH3/G3.tif]

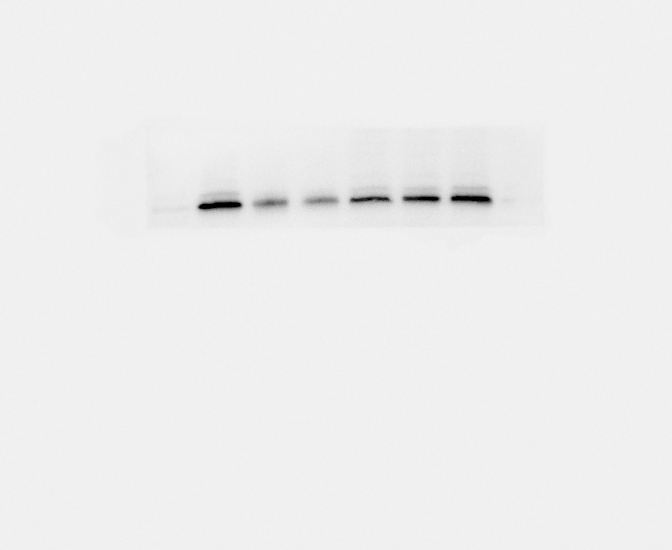

Supplement: Supplemental Information 2 [file peerj-11-16255-s002.zip › WB/TH/TH1/T1.1.tif]

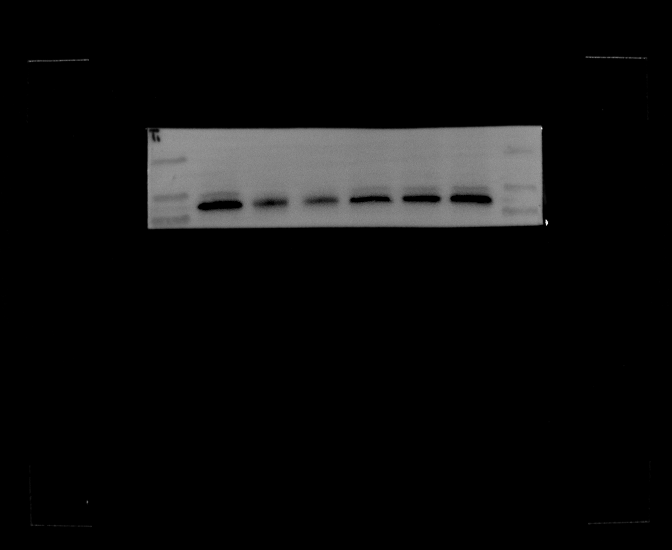

Supplement: Supplemental Information 2 [file peerj-11-16255-s002.zip › WB/TH/TH1/T1.tif]

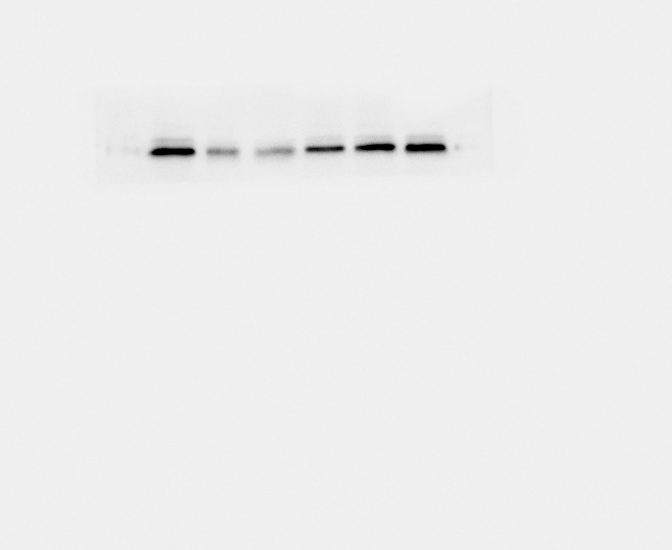

Supplement: Supplemental Information 2 [file peerj-11-16255-s002.zip › WB/TH/TH2/T2.1.tif]

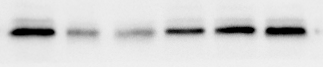

Supplement: Supplemental Information 2 [file peerj-11-16255-s002.zip › WB/TH/TH2/T2.2.tif]

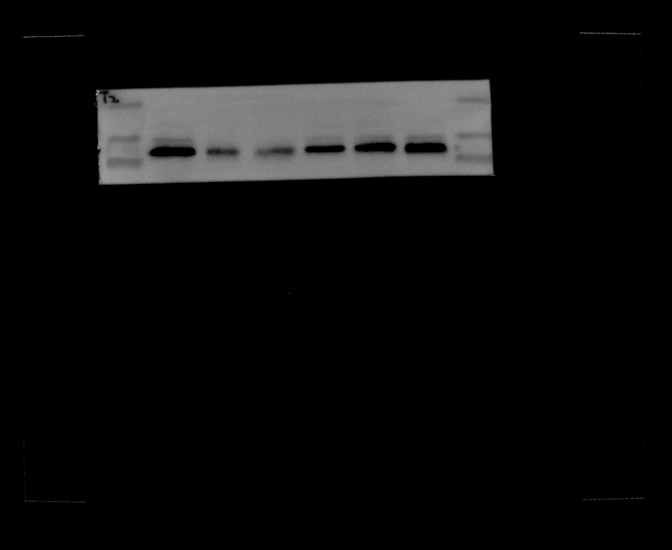

Supplement: Supplemental Information 2 [file peerj-11-16255-s002.zip › WB/TH/TH2/T2.tif]

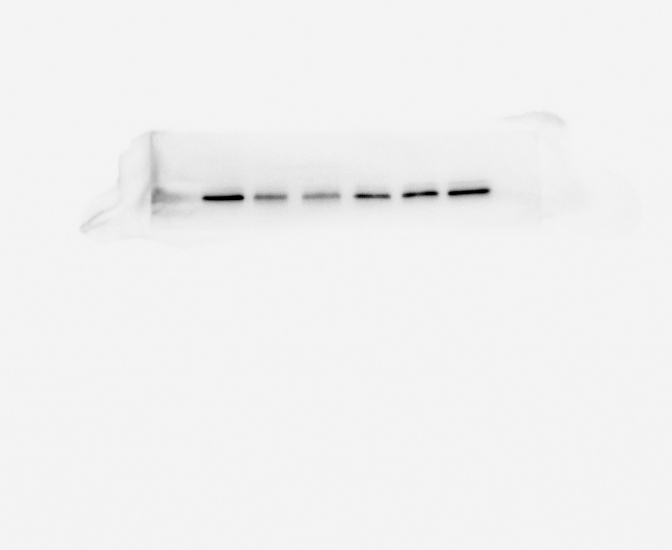

Supplement: Supplemental Information 2 [file peerj-11-16255-s002.zip › WB/TH/TH3/T3.1.tif]

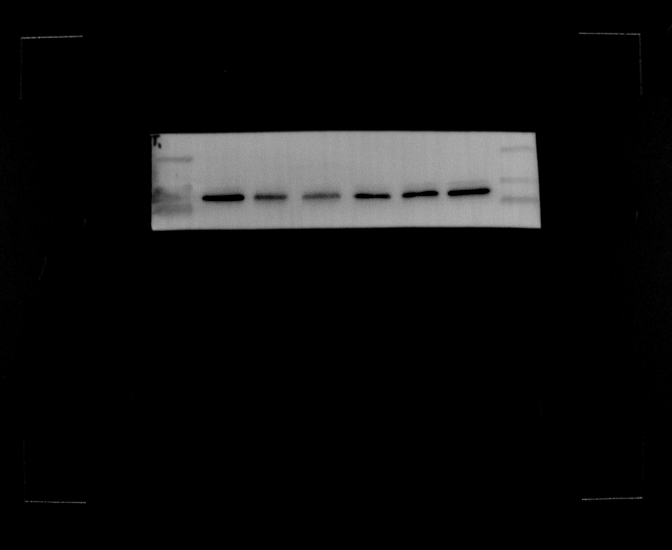

Supplement: Supplemental Information 2 [file peerj-11-16255-s002.zip › WB/TH/TH3/T3.tif]
